# Mentoring in Hospital Settings: A Systematic Review of Guidance, Care, and Professional Development

**DOI:** 10.3390/healthcare14040505

**Published:** 2026-02-15

**Authors:** Giuliana Ventimiglia, Ilaria Setti, Marina Maffoni

**Affiliations:** 1 Department of Brain and Behavioral Sciences, University of Pavia, 27100 Pavia, Italy; giulianaventimiglia@gmail.com (G.V.); ilaria.setti@unipv.it (I.S.); 2 Psychology Unit, Istituti Clinici Scientifici Maugeri IRCCS, Montescano Institute, 27040 Montescano, Italy

**Keywords:** mentoring, mentorship, psychological support, healthcare, systematic review

## Abstract

Background/Objectives: Mentoring is defined as a supportive relationship between an experienced professional (mentor) and a less experienced individual (mentee), influencing skill development, professional confidence, and psychological well-being. This systematic review addresses the question: “Can support from a senior colleague positively impact junior healthcare workers?” Methods: Following PRISMA 2020 guidelines, a systematic literature search was performed (January 2004–December 2024) in Web of Science, PubMed, and Scopus databases, yielding 399 studies. Results: After rigorous screening and quality assessment using the QuADS checklist, 74 studies were included in the final analysis. The reviewed articles span various healthcare fields, including nursing, medicine, and midwifery, utilizing qualitative, quantitative, observational, and mixed-methods approaches. Key findings highlight the mentor’s role in academic and emotional support; fostering clinical and transversal skills such as communication, collaboration, and problem-solving; and enhancing self-efficacy, resilience, and autonomy, particularly during transitional or emotionally demanding periods. Challenges identified include the need for inclusive environments and standardized mentoring models. Conclusions: Overall, mentoring supports the professional and personal growth of junior healthcare professionals and contributes positively to training quality and clinical work. However, issues regarding equitable access, program standardization, and the need for further research to establish consolidated guidelines remain.

## 1. Introduction

Mentoring, defined as a dynamic relationship whereby a more experienced and competent individual (mentor) actively supports the development of a less experienced person (mentee), has emerged as a fundamental component in the education and professional growth of junior healthcare professionals [1]. It is a supportive relationship focused on long-term professional growth, identity formation, and holistic support within a particular professional context. In contrast, supervision is primarily oriented toward performance evaluation, task oversight, and ensuring adherence to clinical or educational standards, while tutoring typically targets the acquisition of specific knowledge or technical skills in a more short-term and content-focused manner. Particularly, within clinical settings characterized by complex, novel challenges, mentoring plays a critical role in bridging theoretical knowledge and practical skills, fostering decision-making abilities, relational competencies, and professional identity formation [1]. Therefore, mentoring is a relational and longitudinal process that complements supervision or tutoring in healthcare education and practice.

The mentoring relationship has long been studied, but only recently has it gained widespread recognition as a key factor in professional competency development, especially in healthcare contexts demanding adaptability and resilience. Indeed, a growing body of theoretical work has sought to unpack the multifaceted nature of mentoring, highlighting its roles in professional development, identity formation, and psychological well-being. Among these, Kram’s Mentor Role Theory distinguishes between career and psychosocial functions of mentoring [2], the Job Demands–Resources (JD-R) model frames mentoring as a key job resource that buffers work demands [3,4], and Self-Determination Theory (SDT) emphasizes the importance of autonomy, competence, and relatedness for motivation and well-being [5]. Overall, effective mentoring programs have demonstrated improvements in academic performance and motivation, and significant psychological benefits that help reduce stress and anxiety, and prevent burnout among trainees [6,7]. Adequate preparation of mentors for the emotional and psychological dimensions of this role enhances the relational support they provide, benefiting both mentees and mentors themselves by fostering their reflective, communicative, and problem-solving skills [6,8].

Mentoring models are diverse, including peer mentoring, group mentoring, and mosaic mentoring, each adapted to specific educational needs and organizational contexts. Peer mentoring offers relatable support for trainees through mentors with slightly more experience, while group mentoring exposes mentees to multiple perspectives, thus enriching learning and promoting inclusivity [9,10]. Mosaic mentoring involves a network of mentors with varied expertise, particularly beneficial for underrepresented groups, by providing culturally sensitive and multilateral guidance [11,12].

In demanding clinical environments such as hospitals, where experiences can be emotionally taxing, mentoring extends beyond skill transmission to include psychological support, contributing to enhanced well-being, stress management, and sense of belonging within professional teams [1,13]. Importantly, mentoring has been shown to influence career choices positively by encouraging pursuit of challenging specialties and reducing attrition by providing stable and empathetic guidance during transitions, such as from academic study to clinical practice [14,15].

Despite increased scholarly interest, the mentoring literature is characterized by conceptual and methodological heterogeneity, with varying definitions, roles, goals, and assessment tools across disciplines and settings [16,17]. Emerging mentoring formats, including virtual platforms and co-productive, non-hierarchical relationships, broaden the traditional understanding of mentoring but also highlight the need for standardized frameworks and robust evaluation instruments tailored to healthcare environments [14,15].

Previous systematic reviews have examined mentoring in healthcare but reveal key gaps that this study addresses. Specifically, prior reviews have targeted mentoring across select healthcare professional groups, primarily focusing on nursing, medical, allied health professions and social care students or early career professionals. For instance, Guo et al. [18] examined peer mentoring exclusively among undergraduate nursing students, while Ellis et al. [19] focused on medical students and early career physicians, and Juntunen et al. (2024) [20] addressed undergraduate healthcare, social care, and medical students in various healthcare contexts. To our knowledge, no comprehensive synthesis spans all healthcare specialties in hospital settings.

This review fills that gap by providing a multidisciplinary evidence synthesis that emphasizes senior mentoring’s role—regardless of specialty, whether medical, nursing, or other allied health professions —in fostering professional skills and psychological resilience during clinical transitions. Specifically, it addresses the research question (i.e., whether support from a senior colleague positively impacts junior healthcare workers), structured using the PICo framework (Population, Phenomenon of Interest, and Context) appropriate for qualitative and mixed-methods evidence synthesis:

- Population: Junior healthcare professionals (physicians, nurses, midwives, and other allied health professions).
- Phenomenon of Interest: Senior mentoring and its impact on professional and personal growth.
- Context: Hospital and healthcare settings.

Although mentoring can occur across diverse healthcare contexts—including outpatient clinics and community-based services—this study focuses specifically on hospital settings. Hospitals are characterized by high workload, complex interprofessional teams, and intense emotional demands, which may amplify both the need for mentoring and the associated risks. By concentrating on this environment, we aim to examine mentoring within a context where supervision is often formalized, roles are clearly defined, and institutional structures are more visible, thereby facilitating a more in-depth exploration of mentoring dynamics in acute-care systems.

Therefore, this systematic review synthesizes current evidence to examine whether and how senior mentoring supports the professional and personal development of junior healthcare professionals in hospital settings, regardless of their specific role. Although the preponderance of existing literature focuses on nursing and medical education, similar challenges are increasingly recognized within all allied health professions. Distinct from previous reviews that addressed mentoring in specific nursing contexts or focused broadly on educational outcomes [19,20], this review uniquely integrates the psychological well-being dimension within the multidisciplinary hospital setting. Unlike prior work, we synthesize evidence spanning the entire continuum of clinical training—from students to early career professionals—identifying how mentoring acts not only as an educational tool but as a critical buffer against occupational stress in high-intensity clinical environments. Moreover, this review elucidates effective mentoring approaches, highlights benefits and challenges, and proposes directions for future research and practice to strengthen mentoring as a foundational element for developing competent, resilient, and well-supported healthcare professionals.

## 2. Materials and Methods

### 2.1. Study Design and Protocol

This study was designed as a systematic literature review to identify, evaluate, and synthesize available evidence regarding the role of mentoring in the training and professional development of healthcare students, with a specific focus on trainees in hospital settings. A systematic review design was selected over a scoping or realist review because the primary objective was not merely to map the available literature or explore theoretical mechanisms, but to critically appraise the quality of evidence and synthesize specific outcomes regarding the impact of mentoring [21]. This approach allows for a more rigorous assessment of the effectiveness of mentoring interventions in the clinical setting.

A formal protocol was not registered in the PROSPERO database; however, the review followed a pre-defined internal protocol developed by the research team to ensure consistency and transparency.

The review process followed the Preferred Reporting Items for Systematic Reviews and Meta-Analyses (PRISMA) 2020 statement guidelines, ensuring transparency, methodological rigor, and replicability [22]. The PRISMA 2020 framework was selected to improve the reporting quality of the review, adhering to its 27-item checklist and flow diagram requirements. As noted by Pati and Lorusso [23], a systematic review offers a consistent and universally recognized method to minimize bias and enhance the credibility of the research process. This approach was chosen over a traditional narrative review to provide a more objective and comprehensive evaluation of the scientific evidence, thereby creating a solid foundation for decision-making in clinical and academic contexts.

Moreover, this review was guided by the methodological framework of the Joanna Briggs Institute (JBI) for systematic reviews of mixed evidence [24]. This ensures our approach to search, selection, and synthesis adheres to established rigorous standards.

### 2.2. Search Strategy

To comprehensively identify relevant literature, a systematic search was conducted across three major international electronic databases: *Web of Science*, *PubMed*, and *Scopus*, deemed to provide the most robust coverage of the general clinical hospital setting. The search strategy employed a specific string of keywords combined with Boolean operators (AND, OR) to target the population, intervention, and outcomes of interest. The search string used was as follows: *(“mentoring” OR “mentorship” OR “tutoring” OR “tutorship” OR “mentor-mentee relationship” OR “mentor”) AND (“medical student*” OR “psychology student*” OR “nursing student*” OR “nurs* student*” OR “health care student*” OR “healthcare student*” OR “physiotherapy student*” OR “therap* student*” OR “physical therapy student*”) AND (“psychological wellbeing” OR “psychological well-being” OR “mental health”)*. The search strategy combined MeSH terms such as ‘Mentors’, ‘Education, Medical’, and ‘Interprofessional Relations’ with free-text keywords including ‘mentoring’, ‘preceptorship’, and ‘junior health personnel’. These were combined using the Boolean operators OR (within concepts) and AND (between concepts).

We used language (English, Italian, Spanish) and date (January 2004–December 2024) filters directly in the database searches.

### 2.3. Eligibility Criteria

Strict inclusion and exclusion criteria were defined to select high-quality studies relevant to the research question. The time frame for inclusion was set from January 2004 to December 2024, based on the observation by Ulrich et al. [25] that the volume of healthcare research significantly increased after 2004.

The inclusion criteria required studies to focus on support provided by senior professionals to junior operators or trainees. We included both undergraduate students and early career professionals to capture the full continuum of clinical education and professional socialization. Moreover, eligible papers must use experimental designs (including quantitative, qualitative, or mixed-methods), be conducted specifically within hospital settings, and be published in peer-reviewed indexed journals. Additionally, full-text articles had to be available in English, Italian, Spanish.

Conversely, the exclusion criteria eliminated studies addressing support for professionals managing territorial or outpatient patients (non-hospitalized contexts) and studies with mixed samples where less than 50% of participants worked in a hospital setting. A threshold of ≥50% hospital-based activity was established to ensure that the primary learning environment was the clinical ward rather than the academic classroom. Articles reporting the perspectives of patients or non-healthcare individuals were also excluded, as were grey literature sources such as editorials, commentaries, theses, and opinion papers. Secondary research, including systematic reviews and meta-analyses, was similarly omitted from the final selection.

Regarding the distinction between mentoring and tutoring, although ‘tutoring’ was included in the initial search string to ensure sensitivity (capturing studies that might use the terms interchangeably), strict conceptual filtering was applied during manual screening. Studies describing purely task-oriented, short-term tutoring interventions without the longitudinal, relational, and holistic support characteristics of mentoring defined in the Introduction were excluded.

These criteria were developed to operationalize the PICo-formulated review question stated in the Introduction, ensuring focus on senior mentoring (Phenomenon of Interest) for junior professionals (Population) in hospital contexts.

### 2.4. Study Selection and Data Extraction

The selection process was documented to ensure reproducibility. All retrieved records were imported into Microsoft Excel for deduplication. Duplicates were identified by exact matching of DOI (primary), followed by title/author fuzzy matching using conditional formatting and SORT/FILTER functions.

Subsequently, title/abstract and full-text screening were conducted independently by two reviewers (VG and MM). Discrepancies were resolved through discussion with a third expert (IS) to prevent arbitrary exclusion and ensure reliability. Inter-rater reliability statistics (e.g., Cohen’s Kappa) were not calculated; instead, we adopted a consensus-based approach typical of qualitative synthesis, where disagreements are resolved through discussion to deepen the interpretative understanding of the data. Only records reaching unanimous agreement proceeded to the following selection stages and were finally included in the review.

Firstly, titles were screened to exclude clearly irrelevant studies based on the established criteria. Abstracts of the remaining records were then reviewed to assess eligibility regarding the study objective, context, and design. Finally, the full texts of potentially eligible articles were retrieved and critically examined to confirm adherence to the inclusion criteria.

Data extraction was conducted using an ad hoc form informed by the research team’s prior experience with systematic reviews of mixed-methods healthcare literature. The form was iteratively refined through team discussion to ensure comprehensive capture of key study characteristics (author, year, design, population, mentoring intervention, outcomes).

### 2.5. Quality Assessment

To evaluate the methodological quality of the included studies, the Quality Assessment with Diverse Studies (QuADS, [26]) tool was employed. This is specifically designed to appraise studies with diverse designs—including qualitative, quantitative, and mixed-methods research—making it particularly suitable for this review. The QuADS tool consists of 13 criteria assessing aspects such as the theoretical rationale, clarity of objectives, appropriateness of the study design, data collection and analysis methods, and discussion of limitations. Each item is scored on a scale from 0 to 3 (where 0 indicates no information and 3 indicates clear and complete description), allowing for a consistent and nuanced evaluation of the evidence strength across methodologically heterogeneous studies.

### 2.6. Data Synthesis

Inductive meta-aggregation followed the JBI guidelines for mixed-methods reviews [24]. For qualitative studies, findings, participant quotes, and author interpretations were directly extracted from results/discussion sections. Quantitative data were transformed into qualitative findings through a two-step process: (1) abstraction—extracting key results, statistics, and their interpretations from Results/Discussion sections; (2) qualitativization—reformulating these as textual statements. That is, quantitative results were “qualitized” by converting statistical significance into narrative statements of “positive/negative association” before aggregation. These analyses were conducted independently by two reviewers (G.V. and M.M.). Similar-meaning statements were grouped into categories, which were then aggregated into analytical themes through team discussion, with discrepancies resolved by I.S.

## 3. Results

### 3.1. Search Results and Study Selection

The systematic literature search, conducted in adherence to the PRISMA 2020 guidelines [22], identified a total of 399 records from three major databases: Scopus (n = 182), PubMed (n = 114), and Web of Science (n = 103). Following the initial removal of 144 duplicates, 255 unique records remained for screening.

In the first stage, title screening led to the exclusion of 49 articles: 32 were unrelated to the review topic, 2 focused exclusively on the patient perspective rather than that of professionals, and 15 were set in non-hospital contexts. Subsequently, the abstracts of the remaining articles were reviewed, resulting in the exclusion of 46 additional records. Reasons for exclusion at this stage included irrelevance to the topic (n = 12), focus on patient perspectives (n = 10), classification as grey literature (e.g., editorials, theses, opinion papers) (n = 24).

**Figure 1:**
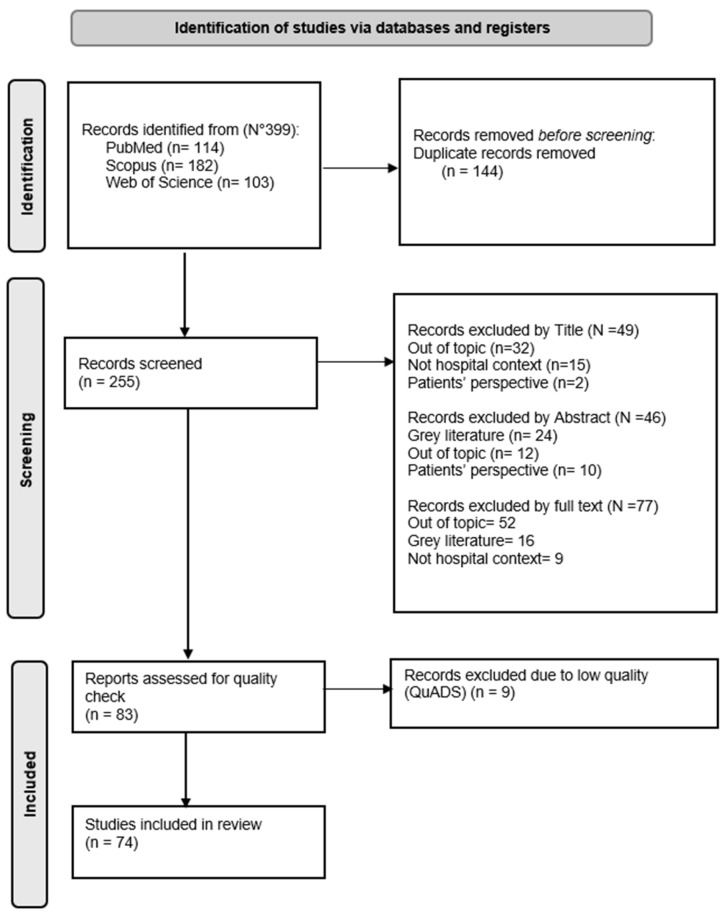
PRISMA 2020 flow diagram.

The remaining articles underwent a full-text review: 52 were outside the specific research scope, 9 were conducted in non-hospital settings, and 16 were grey literature. The final 83 eligible articles were then subjected to a qualitative assessment using QuADS tool ([26], See Supplementary Material S1. References of excluded articles are reported in notes at the bottom of the table) to exclude low-quality papers. Each study was independently evaluated across 13 criteria. Nine studies that demonstrated significant methodological deficiencies (scoring ≤ 1 on multiple dimensions [26]) were excluded. Consequently, 74 studies [9,10,11,12,14,16,18,27,28,29,30,31,32,33,34,35,36,37,38,39,40,41,42,43,44,45,46,47,48,49,50,51,52,53,54,55,56,57,58,59,60,61,62,63,64,65,66,67,68,69,70,71,72,73,74,75,76,77,78,79,80,81,82,83,84,85,86,87,88,89,90,91,92,93,94] satisfied all quality and eligibility criteria and were included in the final systematic review. The entire selection process is illustrated in the PRISMA flow diagram (Figure 1).

### 3.2. Study Characteristics

The included studies demonstrated high heterogeneity regarding geographical origin and methodological approach, underscoring the global relevance and transversal nature of mentoring in healthcare. Geographically, the majority of research originated from Anglophone countries, where mentoring is a well-established professional development strategy: 22 studies were conducted in the United States, 16 in the United Kingdom, seven in Canada, and five in Australia. Contributions were also identified from Continental Europe (Spain, France, Finland, Netherlands, Hungary, Germany, Sweden, Ireland), Asia (India, Japan, Iran, Taiwan, Turkey), and Latin America (Brazil). One study was a transnational collaboration between the United States and Canada.

Methodologically, the review encompassed a diverse range of study designs. A significant portion (n = 33) employed qualitative methods, utilizing semi-structured interviews, focus groups, or observations to explore the nuances of mentoring relationships. Fifteen studies were descriptive, while eight utilized quantitative designs with structured questionnaires and statistical scales to measure outcomes such as satisfaction and performance. Nine studies adopted a mixed-methods approach, integrating qualitative depth with quantitative robustness. The remaining studies included observational (n = 3), longitudinal (n = 3), and pilot or methodological research. Regarding healthcare specialties, the most represented fields were medicine (23 studies) and nursing (18 studies), followed by contributions from midwifery, psychiatry, and clinical psychology (24 studies).

### 3.3. Thematic Analysis

**Figure 2:**
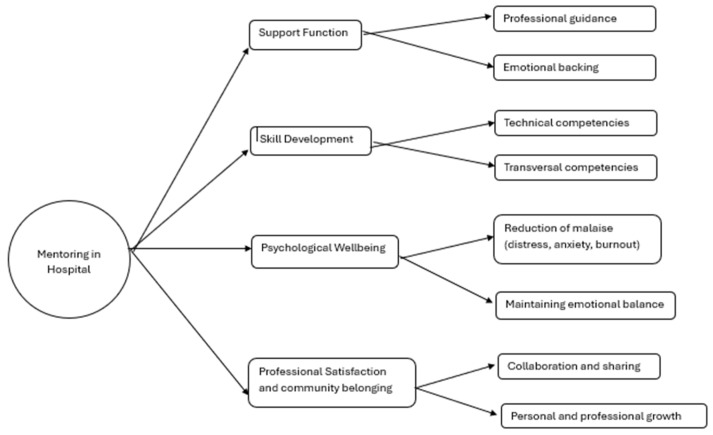
Thematic map of findings. Note. Thematic map of synthesized findings demonstrating the interconnectedness of the four identified themes: Support Function, Skill Development, Psychological Well-being, and Professional Satisfaction and Community Belonging. The central role of the mentor (center) radiates outward to influence both technical competence and psychosocial health, illustrating the holistic impact of mentoring on junior healthcare professionals.

Meta-aggregation (Section 2.6) of transformed findings from all studies provided a coherent picture of the value and impact of mentoring in hospital settings (Supplementary Material S2, [9,10,11,12,14,16,18,27,28,29,30,31,32,33,34,35,36,37,38,39,40,41,42,43,44,45,46,47,48,49,50,51,52,53,54,55,56,57,58,59,60,61,62,63,64,65,66,67,68,69,70,71,72,73,74,75,76,77,78,79,80,81,82,83,84,85,86,87,88,89,90,91,92,93,94]). Specifically, the inductive analysis yielded four main themes: Support Function, Skill Development, Psychological Well-being, and Professional Satisfaction and Community Belonging. As illustrated in Figure 2, these themes are not isolated but dynamically interact; for instance, mentors’ support fosters both professional and emotional guidance.

#### 3.3.1. Support Function

A primary theme identified was the function of support, encompassing both professional guidance and emotional backing. Numerous studies [18,31,33,35,38,39,40,42,45,47] reported that mentees viewed their mentors as stable reference figures capable of offering encouragement and active listening during periods of uncertainty. This support was found to be particularly critical during transitional phases, such as the shift from academic environments to clinical practice, and in high-pressure emotional contexts.

#### 3.3.2. Skill Development

Mentoring was centrally linked to the development of both technical and transversal competencies. Many studies [29,38,43,45,52,62,68,77] documented improvements in specific clinical skills, problem-solving abilities, and complex case management. Parallel to technical growth, studies highlighted enhancements in communication skills, interpersonal relationship management, and interdisciplinary collaboration [12,27,30,38,45,46,56]. This process was often accompanied by increased self-confidence and self-efficacy, positively influencing decision-making autonomy and resilience in facing unforeseen situations [47,56,71,72,80]. The mentor–mentee relationship frequently emerged as a bidirectional learning process, where mentors also refined their leadership and communication capabilities.

#### 3.3.3. Psychological Well-Being

Several studies emphasized the protective effect of mentoring on psychological well-being, noting reductions in stress, anxiety, and burnout [18,56,62,64,88]. In some instances, mentoring provided a “safe space” for sharing difficulties and reflecting on critical experiences without fear of judgment [10,11,63,75,83,84,85,86,87,88,89,90,91,92,93]. This dimension of well-being was closely tied to the ability to maintain emotional balance and redirect energy when facing obstacles.

#### 3.3.4. Professional Satisfaction and Community Belonging

A further key theme was the enhancement of professional satisfaction and a sense of belonging to the healthcare community. Mentees reported that mentoring fostered stronger ties with the organization and the team, facilitating integration into clinical settings [29,34,45,54,56,71,74,75,76,77]. Additionally, mentoring was found to drive broader cultural changes within healthcare organizations by promoting collaboration and the sharing of best practices [38,39,43,47,65,66,67]. In specific contexts, this contributed to improvements in the overall quality of care and user satisfaction [43,44,45,46,54,60,72,77,78,92]. Overall, the results indicate that mentoring extends beyond technical training, acting as a catalyst for personal, professional, and organizational growth.

## 4. Discussion

One of the central findings emerging from this review is that the effectiveness of mentoring cannot be attributed to a single factor, but rather to a complex interplay between the quality of the relationship, institutional support, and specific mentor training. This evidence confirms previous literature describing mentoring as a bidirectional relational process capable of influencing both academic and personal domains [18].

Overall, our findings can be interpreted through several theoretical lenses that elucidate the mechanisms linking mentoring to professional and psychological outcomes. Consistent with Kram’s Mentor Role Theory, the participants reported benefits that align with both career functions (e.g., skill acquisition, professional guidance) and psychosocial functions (e.g., emotional support, role modeling) [2]. The explicit engagement with these psychosocial functions suggests that mentoring provides a protective space for identity formation, which is critical during transition phases in healthcare careers.

Furthermore, regarding psychological well-being, the results support the Job Demands–Resources (JD-R) model [3,4]. In the high-demand context of healthcare—characterized by emotional exhaustion and heavy workloads—mentoring acts as a vital job resource. By providing social support and constructive feedback, mentoring buffers the physiological and psychological costs of these job demands, thereby reducing the risk of burnout and enhancing engagement. Additionally, from the perspective of Self-Determination Theory (SDT, [5]), the mentoring relationship likely fosters psychological well-being by satisfying the basic psychological needs for competence (through professional development) and relatedness (through the mentor–mentee bond), which are prerequisites for intrinsic motivation and mental health in clinical settings.

Thus, our findings align with the main theoretical frameworks introduced. The emotional support identified in our themes maps directly to the ‘psychosocial functions’ of Kram’s theory [2] and the ‘relatedness’ need in Self-Determination Theory [5]. Furthermore, the role of mentors in reducing burnout validates the Job Demands–Resources (JD-R) model [3,4], positioning mentoring as a key ‘job resource’ that buffers the high demands of hospital care.

Specifically, this review highlights that mentor availability, empathy, and the capacity to provide constructive feedback are essential conditions for creating a meaningful experience, consistent with findings by Nebhinani et al. [42] and Abrams et al. [67].

Specifically, mentoring has a critical role in fostering psychological safety which allows junior professionals to ask questions, admit errors, and seek feedback without fear of negative consequences. Our findings suggest that effective mentoring creates a ‘safe container’ for learning, which is essential for mitigating burnout and enhancing professional confidence in high-pressure hospital environments.

A comparison with international literature reveals interesting divergences. For instance, Gardner [95], reflecting on doctoral nursing education in the United States, highlights that the mentoring relationship plays a crucial role not only in emotional and professional support but also in the construction of academic and research identity. Although this aspect is considered less prominent in the European studies included in this review, it represents a relevant insight, demonstrating how mentoring can assume different meanings depending on the context and level of training.

More recent systematic reviews provide further nuance. Ellis et al. [19] demonstrated that structured mentoring, coaching, and peer-support programs are associated with improved well-being and reduced burnout among physicians, reinforcing the protective role of mentoring in high-stress clinical environments. In line with these results, our review suggests that the psychological support and guidance provided through the mentor–mentee relationship can buffer occupational stress and enhance resilience.

Similarly, Juntunen et al. (2025) [20] synthesized qualitative evidence on the competencies required for mentoring interprofessional students in clinical practice, emphasizing skills such as facilitating reflective learning, fostering collaboration, and supporting professional identity development. Overall, these pieces of literature suggest that mentoring is not limited to personal and relational growth but can also serve as a fundamental vehicle for developing innovative professional skills, which are now indispensable for clinical practice.

From a geographical perspective, it is observed that studies from non-European contexts (such as India or the United States) place greater emphasis on the educational dimension and academic career construction. In contrast, European and South American studies more strongly highlight the function of psychological support and guidance through difficulties [66]. This difference likely reflects the varying weight that different educational and healthcare systems attribute to the technical versus the relational dimensions of mentoring.

A further critical element concerns risk factors. In healthcare settings, several potential risks can undermine the effectiveness and safety of mentoring relationships. These include excessive workload and time constraints on mentors, power imbalances between senior mentors and junior mentees, mismatched expectations about the goals of mentoring, and inadequate psychological safety, which may discourage open disclosure of stress or errors. Systematic and scoping reviews in graduate medical education and nursing highlight that poorly structured programs, lack of mentor training, and insufficient institutional oversight can amplify these risks, sometimes leading to disillusionment or even unprofessional behavior [33,96,97]. Our review confirms that barriers such as lack of time, organizational difficulties, and power dynamics can compromise the trust relationship, as discussed by Nebhinani et al. [42]. The hierarchical nature of healthcare can introduce complex power dynamics into the mentoring relationship. If not managed carefully, these dynamics may inhibit open communication. Future mentorship programs must therefore include specific training on navigating hierarchy to ensure the relationship remains supportive rather than directive. However, the novelty emerging here is that such difficulties should not be viewed exclusively as obstacles, but as useful indicators for understanding the organizational conditions necessary to render programs truly effective. In this sense, limitations become opportunities to rethink mentoring as a more structured device, capable of integrating emotional support, clinical skill development, and professional growth. Ultimately, the results of this review appear largely consistent with established literature but also offer new perspectives. On one hand, they confirm the centrality of the relationship and institutional support, as clearly defined meeting schedules, shared expectations, and institutional support can reduce key risk factors while preserving the benefits of mentoring for professional development and psychological well-being [97]. On the other hand, they suggest that mentoring must be conceived as a flexible tool, capable of adapting to different educational levels, specific cultural contexts, and the new challenges facing healthcare professions, such as digitalization and the increasing complexity of required competencies.

### 4.1. Strengths and Limitations

This systematic review has several notable strengths. First, it followed a rigorous methodological approach guided by the PRISMA 2020 statement [22], ensuring a transparent, reproducible, and comprehensive selection process. The search strategy was extensive, covering three major international databases (Web of Science, PubMed, Scopus) and including a broad range of healthcare disciplines (medicine, nursing, psychology, and physiotherapy), which enhances the generalizability of the findings to the wider healthcare context. Furthermore, the use of the QuADS tool [26] allowed for a consistent evaluation of studies with heterogeneous designs—qualitative, quantitative, and mixed-methods—providing a nuanced assessment of the evidence quality. The inclusion of studies from diverse geographical regions (Europe, North America, Asia, and South America) also strengthens the review by offering a global perspective on mentoring practices, highlighting both cross-cultural commonalities and context-specific differences.

However, the study also presents some limitations. First, despite the rigorous search strategy, limiting the inclusion criteria to articles published in English, Italian, Spanish may have led to the exclusion of relevant studies published in other languages, potentially introducing a language bias. However, these languages were selected based on the authors’ proficiency to ensure accurate data extraction. Similarly, while the exclusion of specialized databases such as CINAHL and PsycINFO may introduce some selection bias, the selected databases (PubMed, Scopus, and Web of Science) were deemed to provide the most robust coverage of the general clinical hospital setting central to our research question. Second, the significant heterogeneity of the included studies—ranging from varying mentoring definitions and models to different assessment tools and outcomes—made it challenging to perform a quantitative meta-analysis. Consequently, the findings are presented as a narrative synthesis, which, while rich in detail, limits the ability to statistically quantify the overall impact of mentoring interventions. In addition, given the qualitative, thematic nature of our review, in which the focus was on interpretive analysis of mentoring experiences and their practical implications, we did not calculate formal inter-rater reliability statistics (e.g., Cohen’s kappa) for the screening and selection process. While disagreements were resolved through discussion and consensus, the absence of a quantified measure of agreement may reduce the transparency of the selection phase compared with reviews that explicitly report such indices [98]. Again, regarding quality assessment, the decision to exclude studies with a QuADS score of ≤1 was intended to preserve the methodological reliability of the synthesis by removing sources with critically insufficient reporting. However, we acknowledge this threshold constitutes a selection bias, as it may have led to the omission of studies that possessed valid field insights but lacked rigorous academic reporting standards. Third, many included studies employed cross-sectional designs or relied on self-reported measures of satisfaction and well-being, which may be subject to social desirability bias and do not allow for causal inferences regarding the long-term efficacy of mentoring. Additionally, although the review identified key themes such as support and skill development, the variability in how “mentoring” is operationalized across institutions suggests that caution is needed when directly comparing results across different healthcare settings. A further limitation is that the study focuses exclusively on hospital settings, which may limit the transferability of our findings to outpatient or community-based contexts where mentoring may occur in more informal or decentralized ways. Again, while this study includes participants from various stages of professional development, it was designed to assess general trends within the clinical setting. Future studies should aim to explicitly distinguish and compare outcomes between undergraduate students, postgraduate trainees, and early career professionals to better understand group-specific dynamics.

Finally, our synthesis remains largely narrative and does not systematically compare the strength of effects across different mentoring models or healthcare disciplines. In addition, it should be also noted that the theoretical frameworks (Kram’s Mentor Role Theory [2], JD-R [3,4], and SDT [5]) were applied retrospectively to interpret the synthesized findings rather than to guide the initial systematic search and data extraction. While this inductive approach was chosen to minimize confirmation bias and allow themes to emerge naturally from the data, it may have limited the specific theoretical depth compared to a purely deductive review designed explicitly to test these models. In addition, we did not perform a formal analysis of regional differences, so the geographical distribution of studies is presented primarily to illustrate the global scope of the literature rather than to identify meaningful regional patterns.

### 4.2. Practical Implications

The data collected in this review confirm that mentoring extends beyond the transmission of technical skills to play a broader role in sustaining emotional well-being, reducing stress and burnout, strengthening self-efficacy, and fueling motivation.

These findings have significant practical implications, as evidenced by the work of Bellodi and Dolhnikoff [52], who investigated the experiences of medical students with performance difficulties. The authors demonstrate how a structured academic mentoring program can offer concrete support to these students, addressing both educational and emotional needs. In this sense, mentoring reveals itself as an instrument of equity, capable of reducing the risk of exclusion and supporting those in situations of greater vulnerability. These data underscore the practical importance of developing personalized programs, supported by institutions, that can genuinely respond to the needs of the student population.

Furthermore, the review draws attention to the accessibility and inclusivity of mentoring. The study by Gerk et al. [63], conducted among medical students in Brazil, highlighted how gender discrimination influences career aspirations and conditions access to mentoring figures. Not all students have equal opportunities to benefit from this tool; without targeted interventions, there is a risk that inequalities may be amplified rather than reduced. This finding calls for the design of equitable mentoring models that are sensitive to diversity and capable of breaking down barriers related to gender and other forms of discrimination.

Thus, to translate these findings into practice, healthcare institutions should formalize mentoring programs by establishing clear objectives and allocating protected time, rather than relying on informal support. Crucially, mentors require specific training focused on soft skills and psychological safety, ensuring they are equipped to support personal growth beyond technical clinical expertise. Finally, it is vital to explicitly distinguish the developmental role of a mentor from the evaluative role of a clinical supervisor to prevent role ambiguity and conflict.

## 5. Conclusions

Mentoring represents a central instrument for accompanying healthcare students along their educational and professional paths. It emerges as a relational and educational device with great potential, yet its effectiveness depends on how it is conceived, structured, and made accessible. The practical implications indicate a need for targeted programs capable of supporting even the most fragile students, while future perspectives urge the construction of inclusive pathways oriented toward social justice. Only in this way can mentoring fully express its transformative function, contributing to individual growth, the strengthening of professional communities, and the improvement of healthcare and educational systems. Therefore, the main implication for healthcare education policy is that structured mentoring programs should be formally integrated into postgraduate training curricula in hospital settings to systematically support the professional development and psychological well-being of junior healthcare workers.

## Data Availability

Raw data supporting the conclusions of this article will be made available by the authors, under reasonable request.

## Supplementary Materials

The following supporting information can be downloaded at: https://www.mdpi.com/article/10.3390/healthcare14040505/s1: Supplementary Material S1 (QuADS table); Supplementary Material S2 (Table of studies included in this review).

## Abbreviations

The following abbreviations are used in this manuscript:

PRISMA	Preferred Reporting Items for Systematic Reviews and Meta-Analyses
QuADS	Quality Assessment with Diverse Studies

## References

[1] Elce Y. (2021). The Mentor-Mentee Relationship, Addressing Challenges in Veterinary Medicine Together. The Veterinary clinics of North America. Small Anim. Pract..

[2] Kram K.E. (1985). Mentoring at Work: Developmental Relationships in Organizational Life.

[3] Demerouti E., Bakker A.B., Nachreiner F., Schaufeli W.B. (2001). The job demands-resources model of burnout. J. Appl. Psychol..

[4] Bakker A.B., Demerouti E. (2007). The Job Demands-Resources model: State of the art. J. Manag. Psychol..

[5] Ryan R.M., Deci E.L. (2000). Self-determination theory and the facilitation of intrinsic motivation, social development, and well-being. Am. Psychol..

[6] Altonji S.J., Baños J.H., Harada C.N. (2019). Perceived Benefits of a Peer Mentoring Program for First-Year Medical Students. Teach. Learn. Med..

[7] Sutkin G., Littleton E.B., Kanter S.L. (2015). How surgical mentors teach: A classification of in vivo teaching behaviors part 2: Physical teaching guidance. J. Surg. Educ..

[8] Biber D., Rothman R. (2023). Mental health literacy training for college female peer mentors: A pilot study. High. Educ. Ski. Work-Based Learn..

[9] Fournier M., Tourian L. (2020). A peer mentoring initiative across medical residency programs. Ment. Health Soc. Incl..

[10] Fokuo J.K., Goldrick V., Rossetti J., Wahlstrom C., Kocurek C., Larson J., Corrigan P. (2017). Decreasing the stigma of mental illness through a student-nurse mentoring program: A qualitative study. Community Ment. Health J..

[11] Flanigan T.P., Payne N., Simmons E., Hyde J., Sly K., Zlotnick C. (2009). Mentoring per la diversità nell’HIV/AIDS per rafforzare la capacità di ricerca. Am. J. Public Health.

[12] De Jong G., Meijer E., Schout G., Abma T. (2018). Involving undergraduate nursing students in participatory health re-search: Implications from the Netherlands. J. Prof. Nurs..

[13] King C.A., Gipson P.Y., Arango A., Foster C.E., Clark M., Ghaziuddin N., Stone D. (2018). LET’s Connect community mentoring program for youths with peer social problems: Preliminary findings from a randomized effectiveness trial. J. Community Psychol..

[14] Curran M.A., Black M., Depp C.A., Iglewicz A., Reichstadt J., Palinkas L., Jeste D.V. (2015). Barriere e fattori facilitanti percepiti per una carriera accademica in geriatria: Prospettive degli studenti di medicina. Acad. Psychiatry.

[15] Dederichs M., Weber J., Muth T., Angerer P., Loerbroks A. (2020). Students’ perspectives on interventions to reduce stress in medical school: A qualitative study. PLoS ONE.

[16] Linnerud S.C.W., Olaussen C., Bjørk I.T. (2024). The Norwegian version of the nursing student mentoring competence instrument (MCI): A psychometric evaluation. Int. J. Nurs. Stud. Adv..

[17] Barry D., Houghton T., Warburton T. (2016). Supportare gli studenti nella pratica: Leadership. Nurs. Stand..

[18] Guo K.L., Isaacs A.N., Ning X., Denson L.A., Chapman R. (2018). Peer mentoring and peer support programs for undergraduate nursing students: A systematic review. Nurse Educ. Today.

[19] Ellis M.R., Wilson G., Nulan E., Day M.A., McElroy J.A. (2024). Mentoring, coaching and peer-support programs promoting well-being for physicians: A systematic review. Med. Res. Arch..

[20] Juntunen J., Tuomikoski A.M., Pramila-Savukoski S., Kaarlela V., Keinänen A.L., Kääriäinen M., Mikkonen K. (2025). Healthcare professionals’ experiences of required competencies in mentoring of interprofessional students in clinical practice: A systematic review of qualitative studies. J. Adv. Nurs..

[21] Munn Z., Peters M.D.J., Stern C., Tufanaru C., McArthur A., Aromataris E. (2018). Systematic review or scoping review? Guidance for authors when choosing between a systematic or scoping review approach. BMC Med. Res. Methodol..

[22] Page M.J., McKenzie J.E., Bossuyt P.M., Boutron I., Hoffmann T.C., Mulrow C.D., Shamseer L., Tetzlaff J.M., Akl E.A., Brennan S.E. (2021). The PRISMA 2020 statement: An updated guideline for reporting systematic reviews. BMJ.

[23] Pati D., Lorusso L.N. (2018). How to write a systematic review of the literature. Health Environ. Res. Des. J..

[24] Aromataris E., Lockwood C., Porritt K., Pilla B., Jordan Z. (2024). JBI Manual for Evidence Synthesis.

[25] Ulrich R., Zimring C., Quan X., Joseph A., Choudhary R. (2004). The role of the physical environment in the hospital of the 21st century: A once-in-a-lifetime opportunity. Concord CA Cent. Health Des..

[26] Harrison R., Jones B., Gardner P., Lawton R. (2021). Quality Assessment with Diverse Studies (QuADS): An appraisal tool for methodological and reporting quality in systematic reviews of mixed- or multimethod studies. BMC Health Serv. Res..

[27] Oates J., Hassan R., Coster S. (2022). We’re giving them the tools: A qualitative study of nursing students working with Recovery College trainers to support student wellbeing. J. Ment. Health Train. Educ. Pract..

[28] Hardy S., Mushore M., Goddard L. (2016). Supporting mental health nursing students in clinical placement through virtual in practice support (VIPS): Innovation adoption and the ‘VIPS’ project. Nurse Educ. Today.

[29] Bates T., Cohan M.E., Bragg D.S.A. (2006). The Medical College of Wisconsin Senior Mentor Program: Experience of a lifetime. J. Nurs. Educ..

[30] Miles L.W., Mabey L., Leggett S.A., Stansfield K. (2014). Teaching communication and therapeutic relationship skills to baccalaureate nursing students: A peer mentorship simulation approach. J. Nurs. Educ. Pract..

[31] Veganzones I., Cruz C., Farriols R.P. (2024). Programa de apoyo al estudiantado de Medicina. Educación Médica.

[32] Visnjic A.M., Milosavljevic N.D., Djordjevic G.D. (2009). Stress factors of medical students in Serbia. J. Public Health.

[33] Himmelstein R., Guth S., Enenbach M., Gleason M.M., Stevens H., Glowinski A., Kolevzon A., Martin A. (2022). Psychiatry Match Rates Increase After Exposure to a Medical Student Mentorship Program: A Multisite Retrospective Cohort Analysis. Acad Psychiatry.

[34] Higgins A., McCarthy M. (2005). Psychiatric nursing students’ experiences of having a mentor during their first practice placement: An Irish perspective. Nurse Educ. Pract..

[35] Axisa C., Nash L., Kelly P., Willcock S. (2020). Psychiatric morbidity, burnout and distress in Australian physician trainees. Aust. Health Rev..

[36] Liu C., Wang L., Qi R., Wang W., Jia S., Shang D., Shao Y., Yu M., Zhu X., Yan S. (2019). Prevalence and associated factors of depression and anxiety among doctoral students: The mediating effect of mentoring relationships on the association between research self-efficacy and depression/anxiety. Psychol. Res. Behav. Manag..

[37] Boardman G., Lawrence K., Polacsek M. (2019). Undergraduate student nurses’ perspectives of an integrated clinical learning model in the mental health environment. Int. J. Ment. Health Nurs..

[38] Vuckovic A., Karlsson B., Sunnqvist C. (2019). Nursing students’ experiences of support during clinical practice in psychiatric care: An interview study. Nurse Educ. Pract..

[39] Roldán Merino J., Miguel Ruiz D., Roca Capara J. (2019). Perceptions of nursing students on personal tutoring: A qualitative study. Nurse Educ. Today.

[40] Adams K., Hussain N., Farrow M., Jones S. (2024). Personal academic tutors (PATs): A student perspective. Curr. Pharm. Teach. Learn..

[41] Guse J., Heinen I., Kurre J., Mohr S., Bergelt C. (2020). Perception of the study situation and mental burden during the COVID-19 pandemic among undergraduate medical students with and without mentoring. GMS J. Med. Educ..

[42] Nebhinani N., Jagtiani A., Chahal S. (2020). Perception of medical students for faculty and peer mentorship program: An exploratory study from India. J. Indian Assoc. Child Adolesc. Ment. Health.

[43] Rastegar Kazerooni A., Amini M., Tabari P., Moosavi M. (2020). Peer mentoring for medical students during the COVID-19 pandemic via a social media platform. Med. Educ..

[44] Maplethorpe F., Dixon J., Rush B. (2020). 2014. Participation in clinical supervision (PACS): An evaluation of student nurse clinical supervision facilitated by mental health service users. Nurse Educ. Pract..

[45] Schwind J.K., Lindsay G.M., Coffey S., Morrison D., Mildon B. (2014). Opening the black-box of person-centred care: An arts-informed narrative inquiry into mental health education and practice. Nurse Educ. Today.

[46] Fuentes Pumarola C., Ballester Ferrando D., Gelabert Vilella S., Bosch Farré C., Malagón Aguilera M.C., Rascón Hernán C., Bonmatí Tomàs A., Fernández Peña R. (2016). Nursing student and professor perceptions and assessments of the achievement of practicum competencies: A mixed method approach. Nurse Educ. Today.

[47] Sherman J., Kalvas L.B., Schlegel E.C. (2023). Navigating the turbulent seas: Experiences of peer mentorship on the journey to becoming a nurse scholar. Nurse Educ. Today.

[48] Jack K., Hamshire C., Harris W.E., Langan M., Barrett N., Wibberley C. (2018). My mentor didn’t speak to me for the first four weeks: Perceived Unfairness experienced by nursing students in clinical practice settings. J. Clin. Nurs..

[49] Kayama M., Gregg M.F., Asahara K., Yamamoto-Mitani N., Okuma K., Ohta K., Kinoshita Y. (2013). Mentoring doc-toral students for qualitative research: Interviews with experienced nursing faculty in Japan. J. Nurs. Educ..

[50] Wood S. (2010). Mental health nursing students’ views of pre-registration nursing. Nurs. Stand..

[51] Habib A.M., Yu V., Yu M., Levi J.R., Gudis D.A., Overdevest J. (2024). Medical students’ perspectives on how COVID-19 has impacted their otolaryngology educational experience: A nationwide survey study. Ear Nose Throat J..

[52] Bellodi P.L., Dolhnikoff M. (2021). Medical students with performance difficulties need broad support: First results of an academic tutoring program. Clinics.

[53] Murray S.C., Williamson G.R. (2009). Managing capacity issues in clinical placements for pre-registration nurses. J. Clin. Nurs..

[54] White D.L., Cartwright J., Lottes J. (2012). Long-term care nurse role models in clinical nursing education: The ECLEPs experience. J. Nurs. Educ..

[55] King L. (2018). Link lecturers’ views on supporting student nurses who have a learning difficulty in clinical placement. Br. J. Nurs..

[56] Sánchez N.F., Rankin S., Callahan E., Ng H., Holaday L., McIntosh K., Poll-Hunter N., Sánchez J.P. (2015). LGBT Trainee and Health Professional Perspectives on Academic Careers--Facilitators and Challenges. LGBT Health.

[57] Fokuo J.K., Maroney M.M., Corrigan P. (2020). Pilot of a consumer based anti-stigma mentorship program for nursing students. J. Public Ment. Health.

[58] Halpain M.C., Jeste D.V., Trinidad G.I., Wetherell J.L., Lebowitz B.D. (2005). Intensive short-term research training for undergraduate, graduate, and medical students: Early experience with a new national-level approach in geriatric mental health. Acad. Psychiatry.

[59] Bechara Secchin L.S., da Silva Ezequiel O., Vitorino L.M., Lucchetti A.L.G., Lucchetti G. (2020). Implementation of a Longitudinal Mentorship Program for Quality of Life, Mental Health, and Motivation of Brazilian Medical Students. Acad. Psychiatry.

[60] Alexander N.L., Sheu J.C., Villagran A.M., Guerrini C.J., Storch E.A. (2022). Impact of the Texas-Wide Premedical Mentoring Program during the COVID-19 pandemic. Bayl. Univ. Med. Cent. Proc..

[61] Ziring D., Danoff D., Grosseman S., Langer D., Esposito A., Jan M.K., Rosenzweig S., Novack D. (2015). How Do Medical Schools Identify and Remediate Professionalism Lapses in Medical Students? A Study of U.S. and Canadian Medical Schools. Acad. Med. J. Assoc. Am. Med. Coll..

[62] Saarikoski M., Warne T., Aunio R., Leino-Kilpi H. (2006). Group supervision in facilitating learning and teaching in mental health clinical placements: A case example of one student group. Issues Ment. Health Nurs..

[63] Gerk A., Campos L., Naus A., Faria I., Buda A.M., Moura C.B., Graner M., Cazumbá M.L., Jean Pierre T.A., Pompermaier L. (2022). Gender discrimination, career aspirations, and access to mentorship among medical students in Brazil. J. Surg. Res..

[64] Lee S.J., Natour A.K., Geevarghese S.K. (2022). Fireside chats: A novel wellness initiative for medical students in the COVID-19 era. Am. Surg..

[65] Guo T., Chowdhury M., Rasouli R., Patel M. (2023). Exploring the effectiveness of a cascading mentorship model in developing CanMEDS competencies in postgraduate medical education: A qualitative interview study among resident mentors at a medical school in Canada. BMJ Open.

[66] King L. (2019). Exploring student nurses’ and their link lecturers’ experiences of reasonable adjustments in clinical placement. Br. J. Nurs..

[67] Abrams R., Sriranjan A., Park S., Coppola W., Ferris M. (2020). Exploring stories of learning and professional development: Interactions between GP personal tutors and medical students. Med. Educ..

[68] Lombardo C., Wong C., Sanzone L., Filion F., Tsimicalis A. (2017). Exploring mentees’ perceptions of an undergraduate nurse peer mentorship program. J. Nurs. Educ..

[69] Kalindjian N., Hourantier C., Ludot M., Gilles de la Londe J., Corcos M., Cadwallader J.S., Moro M.R., Lachal J., Piot M.A. (2023). Experiences of French medical students during their clerkship in adolescent psychiatry: A qualitative study. Eur. Child Adolesc. Psychiatry.

[70] Cust F. (2018). Increasing confidence of first-year student nurses with peer mentoring. Nurs. Times.

[71] West C.H., Rieger K.L., Chooniedass R., Adekoya A.A., Isse A.A.R., Karpa J.V., Waldman C., Peters-Watral B., Chernomas W.M., Scruby L.S. (2018). Enlivening a community of authentic scholarship: A faculty-mentored experience for graduate students at the 2016 Qualitative Health Research Conference. Int. J. Qual. Methods.

[72] Baskaran R., Mukhopadhyay S., Ganesananthan S., Gamage M.P., Dalavaye N., Ng V., Bennett R., Srinivasan S., Sureshkumarnair P., Spencer R. (2023). Enhancing medical students’ confidence and performance in integrated structured clinical examinations (ISCE) through a novel near-peer, mixed model approach during the COVID-19 pandemic. BMC Med. Educ..

[73] Sopher C.J., Adamson B.J., Andrasik M.P., Flood D.M., Wakefield S.F., Stoff D.M., Cook R.S., Kublin J.G., Fuchs J.D. (2015). Enhancing diversity in the public health research workforce: The research and mentorship program for future HIV vaccine scientists. Am. J. Public Health.

[74] Kishore A., DiGiovanni M., Sun K.L., Kolevzon A., Benoit L., Martin A. (2023). Enhancing child and adolescent psy-chiatry recruitment through a medical student mentorship network: A qualitative study. Acad. Psychiatry.

[75] McAllister A., Dickson K., Rangi M., Griffiths L., Dimov S., Reavley N., Knaak S. (2023). Embedding interpersonal stigma resistance into the medical curriculum: A focus group study of medical students. BMC Med. Educ..

[76] Hu W.C.Y., Woodward-Kron R., Flynn E. (2019). Educator as Diagnostician, Judge and Confidant: A positioning analysis of medical student support encounters. Adv. Health Sci. Educ. Theory Pract..

[77] Mumba M.N., Horton A.G., Cole H., Dickson B., Brown W., Parker K., Tice J., Key B., Castillo R., Compton J. (2023). Development and implementation of a novel peer mentoring program for undergraduate nursing students. Int. J. Nurs. Educ. Scholarsh..

[78] Menchetti I., Pham C. (2024). Describing a novel, national, vertical mentorship program for women in emergency medicine across Canada. Can. J. Emerg. Med..

[79] Shashikala M., Naik K., Narayana K., Deepa C. (2022). Continued Mentorship Program for MBBS Students during COVID-19 Pandemic in Virtual Mode: A Questionnaire-based Observational Study. J. Clin. Diagn. Res..

[80] Wareing M., Green H., Burden B., Burns S., Beckwith M.A.R., Mhlanga F., Mann B. (2018). Coaching and peer-assisted learning (C-PAL)—The mental health nursing student experience: A qualitative evaluation. J. Psychiatr. Ment. Health Nurs..

[81] Saukkoriipi M., Tuomikoski A.M., Sivonen P., Kärsämänoja T., Laitinen A., Tähtinen T., Kääriäinen M., Kuivila H.M., Juntunen J., Tomietto M. (2020). Clustering clinical learning environment and mentoring perceptions of nursing and midwifery students: A cross-sectional study. J. Adv. Nurs..

[82] Uchida C., Uchida M. (2017). Characteristics and Risk Factors for Negative Academic Events: A 27-Year Serial Prevalence Study of 9.7 Million Japanese College Students. Prim. Care Companion CNS Disord..

[83] Martin A., Krause R., Chilton J., Jacobs A., Amsalem D. (2020). Attitudes to psychiatry and to mental illness among nursing students: Adaptation and use of two validated instruments in preclinical education. J. Psychiatr. Ment. Health Nurs..

[84] McDonald C., Henderson A., Barlow P., Keith J. (2021). Assessing factors for choosing a primary care specialty in medical students; A longitudinal study. Med. Educ. Online.

[85] Walsh A. (2015). Are new mental nurses prepared for practice?. Ment. Health Rev. J..

[86] Blowers E.J. (2018). An Investigation of Professional Integrity in Pre-registration Nurse Education: A Modified Grounded Theory Research Study. Nurse Educ. Today.

[87] Blatman Z.M., Tang V., Patel M. (2022). Advancing mentorship opportunities of LGBTQ+ youth through a novel cas-cading mentorship and advocacy training model for medical students. Can. Med. Educ. J..

[88] Yen-Ju Lin B., Liu P.C., Ku K.T., Lee C.C. (2019). Adaptation of Medical Students During Clinical Training: Effects of Holistic Preclinical Education on Clerkship Performance. Teach. Learn. Med..

[89] Masaki C.O., Ogbu-Nwobodo L., Santos L.H., Faisel L.B., Lewis A., Soumare A., Quijije N., Ross R.A. (2022). A Virtual Summer Research and Mentorship Program for Underrepresented in Medicine (URiM) Medical Students in Psychiatry. Acad. Psychiatry.

[90] Wang A.H., Lee C.T., Pina V.R. (2022). A virtual peer mentoring intervention for baccalaureate nursing students: A mixed-methods study. J. Prof. Nurs. Off. J. Am. Assoc. Coll. Nurs..

[91] Baillie L., Chadwick S., Mann R., Brooke-Read M. (2013). A survey of student nurses’ and midwives’ experiences of learning to use electronic health record systems in practice. Nurse Educ. Pract..

[92] Polczman L., Jambor M., Gyorffy Z., Purebl G., Vegh A., Girasek E. (2024). A qualitative study of mentors’ perceptions and experiences of a near-peer mentoring program for medical students. Front. Educ..

[93] Mayen S., Roman C., Cermolacce M., Colson S. (2024). The Delphi method to develop a clinical skills assessment tool for advanced practice nurses in psychiatry and mental health. Rech. En Soins Infirm..

[94] Ünsal E., Yalcinkaya T., Dönmez A., Yucel S.C. (2024). A different country, a different language, a different culture… Educational experiences of international nursing students studying in Turkey: A qualitative study. Nurse Educ. Pract..

[95] Gardner J. (2007). A successful doctoral student experience: The importance of mentoring. Coll. Stud. J..

[96] Keinänen A.L., Lähdesmäki R., Juntunen J., Tuomikoski A.M., Kääriäinen M., Mikkonen K. (2023). Effectiveness of mentoring education on health care professionals’ mentoring competence: A systematic review. Nurse Educ. Today.

[97] Abdelmannan D., Buhumaid R., Salman H., Ba Madhaf W.A.A.H., AlRajaby H.M.K., Zary N., Guraya S.S. (2025). A scoping review of mentorship in Graduate Medical Education: A proposed conceptual framework. Front. Med..

[98] Braun V., Clarke V. (2019). Reflective thematic analysis. Qualitative Psychology.

