# Supplementary material for: Mentoring in Hospital Settings: A Systematic Review of Guidance, Care, and Professional Development
Source: Healthcare (Basel). 2026 Feb 15;14(4):505. doi: 10.3390/healthcare14040505 (PMC12941059; doi:10.3390/healthcare14040505)
Supplement: Supplementary file 1 [file healthcare-14-00505-s001.zip › healthcare-4103832-supplementary.pdf]

Supplementary material 1 – QuADS Table

**Quality assessment (Scores: 0-1= low, 2= medium, 3= high)**	Fokuo 2020 [10]	Roldán-Merino J 2019 [39]	Linnerud 2024 [16]	Bates 2006 [29]	Wood 2005 [1e]	Miles 2014 [30]	Barry D 2016 [17]	Hardy 2016 [28]	Veganzoness 2024 [31]	Visnjic 2009 [32]	Himmelstein 2022 [33]	Higgins 2005 [34]	Axisa 2020 [35]	Liu 2019 [36]	Boardman 2019 [37]	Vuckovic 2019 [38]	Robledo-Gil 2018 [2e]	Schindler 2011 [3e]
1. Theoretical or conceptual underpinning to the research	2	2	2	1	1	2	1	2	2	2	2	3	2	2	2	1	2	1
2. Statement of research aim/s	3	3	3	3	2	3	2	3	3	3	3	3	3	3	3	3	1	0
3. Clear description of research setting and target population	3	3	3	2	2	2	1	2	2	3	3	3	3	3	2	3	2	1
4. The study design is appropriate to address the stated research aim/s	2	2	3	2	2	2	0	2	1	3	3	2	3	3	2	2	1	1
5. Appropriate sampling to address the research aim/s	2	2	3	2	1	2	0	1	1	3	3	2	3	3	2	2	0	1
6. Rationale for choice of data collection tool/s	2	2	3	2	1	2	0	2	2	2	3	3	2	2	2	2	1	2
7. The format and content of data collection tool is appropriate to address the stated research aim/s	3	3	3	3	2	3	0	3	2	3	3	1	3	3	2	2	1	1

8. Description of data collection procedure	2	2	3	1	1	2	0	2	1	2	2	2	2	2	2	2	1	2
9. Recruitment data provided	2	1	3	1	1	1	0	1	1	2	2	3	2	2	1	1	1	1
10.Justification for analytic method selected	2	1	3	1	1	2	0	1	1	2	2	3	2	2	1	1	1	1
11. The method of analysis was appropriate to answer the research aim/s	3	2	3	2	2	2	0	2	1	3	3	3	3	3	2	2	2	1
12. Evidence that the research stakeholders have been considered in research design or conduct	2	2	2	2	1	3	1	2	2	1	2	2	1	1	2	2	1	1
13. Strengths and limitations critically discussed	2	2	2	2	1	2	1	2	2	2	2	3	2	2	2	1	1	1
**Global score (high, medium, low quality)**	**2**	**2**	**3**	**2**	**1**	**2**	**0**	**2**	**2**	**2**	**3**	**3**	**2**	**2**	**2**	**2**	**1**	**1**

**Quality assessment (Scores: 0-1= low, 2= medium, 3= high)**	**Adams K 2024 [40]**	**Guse 2020 [41]**	**Nebhinani 2020 [42]**	**Curran 2015 [14]**	**Rastegar Kazerooni 2020 [43]**	**Maplethorpe 2014 [44]**	**Dacha 2018 [4e]**	**Schwind 2014 [45]**	**Fuentes-Pumarola 2016 [46]**	**Sherman 2023 [47]**	**Jack 2018 [48]**	**Scorsolini-Comin 2020 [5e]**	**Kayama M 2013 [49]**	**Wood S. 2010 [50]**	**Habib 2024 [51]**	**Bellodi PL 2021 [52]**	**Murray 2009 [53]**
1. Theoretical or conceptual underpinning to the research	2	2	2	2	2	2	1	2	2	2	2	1	1	1	2	2	2
2. Statement of research aim/s	3	3	3	3	3	3	2	3	3	3	3	2	3	3	3	3	3
3. Clear description of research setting and target population	3	3	2	3	2	3	2	3	3	3	3	2	2	2	3	3	3
4. The study design is appropriate to address the stated research aim/s	2	3	2	2	2	2	2	0	3	3	2	1	2	2	3	2	3
5. Appropriate sampling to address the research aim/s	2	3	2	2	2	2	1	2	3	2	3	1	2	2	3	2	2
6. Rationale for choice of data collection tool/s	2	2	2	2	2	2	2	2	2	2	2	1	2	1	2	2	2
7. The format and content of data collection tool is appropriate to address the stated research aim/s	3	3	2	3	3	3	1	3	3	3	3	1	2	2	3	3	3
8. Description of data collection procedure	2	2	2	2	2	2	1	2	2	2	2	1	1	1	2	2	2
9. Recruitment data provided	2	2	1	2	1	1	1	1	2	1	2	1	1	1	2	2	2

10.Justification for analytic method selected	2	2	2	2	1	1	1	2	2	2	2	1	1	1	2	2	2
11. The method of analysis was appropriate to answer the research aim/s	3	3	2	3	2	2	1	2	3	2	2	1	2	2	3	3	3
12. Evidence that the research stakeholders have been considered in research design or conduct	2	1	2	1	2	2	1	2	2	2	2	2	2	1	2	2	2
13. Strengths and limitations critically discussed	2	2	1	2	2	2	1	2	2	2	2	1	2	2	2	2	2
**Global score (high, medium, low quality)**	**2**	**2**	**2**	**2**	**2**	**2**	**1**	**2**	**2**	**2**	**2**	**1**	**2**	**2**	**2**	**2**	**2**

**Quality assessment (Scores: 0-1= low, 2= medium, 3= high)**	**White 2012 [54]**	**King 2018 [13]**	**Sanchez 2015 [6e]**	**Flanigan 2009 [11]**	**de Jong 2018 [12]**	**Halpain 2005 [58]**	**Secchin 2020 [59]**	**Alexander 2022 [60]**	**Ziring 2015 [61]**	**Saarikoski 2006 [62]**	**Conroy 2022 [7e]**	**Gerk 2022 [63]**	**Guo 2023 [65]**	**King 2018 [13]**	**Abrams 2020 [67]**	**Lombardo 2017 [68]**	**Kalindjian 2023 [69]**	**Vaughn, 2016 [8e]**	**Cust, 2018 [70]**	**West 2018 [71]**
1. Theoretical or conceptual underpinning to the research	2	2	2	2	2	2	2	2	2	2	1	2	2	2	2	2	2	2	2	3
2. Statement of research aim/s	3	3	1	3	3	3	3	3	3	3	1	3	3	3	3	3	3	3	3	3
3.Clear descriptionof research setting and target population	3	3	2	2	3	3	3	3	3	3	2	2	3	3	3	3	3	2	2	3

4. The study design is appropriate to address the stated research aim/s	2	3	1	2	2	2	2	2	3	2	1	1	3	3	3	2	3	1	2	3
5. Appropriate sampling to address the research aim/s	2	2	1	1	2	2	2	2	3	2	1	1	2	2	2	2	2	1	1	2
6. Rationale for choice of data collection tool/s	2	2	1	2	2	2	2	2	2	2	2	1	2	2	2	2	2	1	2	3
7. The format and content of data collection tool is appropriate to address the stated research aim/s	3	3	2	2	3	3	3	3	3	3	1	2	3	3	3	3	3	2	2	3
8. Description of data collection procedure	2	2	1	1	2	2	2	2	2	2	1	1	2	2	2	2	2	1	1	2
9. Recruitment data provided	2	2	2	1	1	2	2	2	2	2	2	1	2	2	2	2	2	1	1	2
10.Justification for analytic method selected	2	2	1	1	1	1	2	2	2	2	1	1	2	2	2	2	2	1	1	2
11. The method of analysis was appropriate to answer the research aim/s	3	3	1	1	2	2	3	3	3	2	2	1	3	3	3	3	3	1	1	3
12. Evidence that the research stakeholders have been considered in research design or conduct	3	2	2	2	3	2	1	2	2	2	1	2	2	2	2	2	2	2	2	3
13. Strengths and limitations critically discussed	2	2	2	2	2	2	2	2	2	2	2	2	2	2	2	2	2	2	2	2
**Global score (high, medium, low quality)**	**2**	**2**	**1**	**2**	**2**	**2**	**2**	**2**	**3**	**2**	**1**	**2**	**2**	**2**	**2**	**2**	**2**	**1**	**2**	**3**

Quality assessment (Scores: 0-1= low, 2= medium, 3= high)	Baskaran R 2023 [72]	Sopher 2015 [73]	Kishore 2023 [74]	McAllister 2023 [75]	Hu 2019 [76]	Yager 2007 [9e]	Mumba 2023 [77]	Menchetti 2024 [78]	Fokuo 2017 [10]	Shashikala 2022 [79]	Wareing 2018 [80]	Saukkoriipi 2020 [81]	Uchida 2017 [82]	Martin 2020 [83]	McDonald 2021 [84]	Walsh 2015 [85]	Blowers 2018 [86]
1. Theoretical or conceptual underpinning to the research	2	2	2	2	3	2	2	2	2	2	2	2	2	3	3	3	3
2. Statement of research aim/s	3	3	3	3	3	3	3	3	3	3	3	3	3	1	1	3	3
3. Clear description of research setting and target population	3	3	3	3	3	2	2	3	3	2	3	3	3	2	2	3	3
4. The study design is appropriate to address the stated research aim/s	2	2	3	3	3	1	2	2	3	2	3	3	3	2	2	2	2
5. Appropriate sampling to address the research aim/s	2	2	2	2	3	1	2	2	2	2	2	3	3	1	2	2	2
6. Rationale for choice of data collection tool/s	2	2	2	2	3	1	2	2	2	2	2	2	2	0	0	3	3
7. The format and content of data collection tool is appropriate to address the stated research aim/s	3	3	3	3	3	2	3	3	3	3	3	3	3	0	0	1	1
8. Description of data collection procedure	2	2	2	3	2	1	2	2	2	2	2	2	2	1	1	2	2
9. Recruitment data provided	2	2	2	2	2	1	2	2	2	1	2	2	3	2	2	3	3

10.Justification for analytic method selected	2	2	2	2	3	1	1	2	2	2	2	2	2	2	2	3	3
11. The method of analysis was appropriate to answer the research aim/s	3	3	3	3	3	1	2	3	3	2	3	3	3	3	3	3	3
12. Evidence that the research stakeholders have been considered in research design or conduct	2	3	2	3	2	2	2	2	2	2	2	1	1	2	2	2	2
13. Strengths and limitations critically discussed	2	2	2	2	2	2	2	2	2	2	2	2	2	3	3	3	3
**Global score (high, medium, low quality)**	**2**	**2**	**2**	**3**	**3**	**1**	**2**	**2**	**2**	**2**	**2**	**2**	**2**	**2**	**2**	**3**	**3**

**Quality assessment (Scores: 0-1= low, 2= medium, 3= high)**	**Blatman 2022 [87]**	**Yen-Ju Lin 2019 [88]**	**Masa ki 2022 [89]**	**Wang 2022 [90]**	**Baillie 2013 [91]**	**Polczman 2024 [92]**	**Fournier 2020 [9]**	**Mayen 2024 [93]**	**Ünsal 2024 [94]**	**Oates 2022 [27]**	**Lee 2022 [64]**
1. Theoretical or conceptual underpinning to the research	3	3	3	3	3	3	3	3	3	3	2
2. Statement of research aim/s	2	0	2	2	3	3	1	2	3	2	2
3. Clear description of research setting and target population	3	2	3	3	2	3	2	3	3	3	3
4. The study design is appropriate to address the stated research aim/s	2	2	2	2	2	3	2	3	2	2	2
5. Appropriate sampling to address the research aim/s	2	2	2	2	2	2	2	2	2	3	2
6. Rationale for choice of data collection tool/s	2	0	2	2	3	3	1	1	2	2	1

7. The format and content of data collection tool is appropriate to address the stated research aim/s	1	0	1	1	1	2	0	1	1	1	2
8. Description of data collection procedure	3	1	3	2	3	2	2	2	2	2	2
9. Recruitment data provided	3	3	2	3	3	3	3	3	3	3	1
10.Justification for analytic method selected	2	3	2	2	3	3	2	3	2	2	2
11. The method of analysis was appropriate to answer the research aim/s	3	3	3	3	3	3	3	3	3	1	2
12. Evidence that the research stakeholders have been considered in research design or conduct	2	2	2	2	2	2	2	2	2	3	3
13. Strengths and limitations critically discussed	3	3	3	3	3	3	3	3	3	1	2
**Global score (high, medium, low quality)**	**2**	**2**	**2**	**2**	**3**	**3**	**2**	**2**	**2**	**2**	**2**

Notes: References of excluded articles after the quality check

1e:Wood, S. (2005). The experiences of a group of pre-registration mental health nursing students. Nurse Education Today, 25(3), 189–196. https://doi.org/10.1016/j.nedt.2004.12.004

2e: Robledo-Gil, T., Guo, X. M., Horien, C., Herrin, M. A., Encandela, J., & Angoff, N. R. (2017). Utilization and Effectiveness of a Peer Advocate Program for Medical Students. Academic Psychiatry, 42(1), 168–170. https://doi.org/10.1007/s40596-017-0790-5

3e: Schindler, V. P. (2010). Using Service-Learning to Teach Mental Health and Research Skills. Occupational Therapy In Health Care, 25(1), 54–64. https://doi.org/10.3109/07380577.2010.519430

4e: Dacha, S., Wang, L., Li, X., Jiang, Y., Philips, G., Keilin, S. A., Willingham, F. F., & Cai, Q. (2018). Outcomes and quality of life assessment after per oral endoscopic myotomy (POEM) performed in the endoscopy unit with trainees. Surgical Endoscopy, 32(7), 3046–3054. https://doi.org/10.1007/s00464-017-6015-x

5e: Scorsolini-Comin, F. (2020). *Mentoring program with nursing students in the context of the COVID-19 pandemic in Brazil*. **Index de Enfermería, 29**(1–2), 19–23. https://scielo.isciii.es/scielo.php?pid=S1132-12962020000100019&script=sci_arttext

6e: Sánchez, N. F., Rankin, S., Callahan, E., Ng, H., Holaday, L., McIntosh, K., Poll-Hunter, N., & Sánchez, J. P. (2015). LGBT Trainee and Health Professional Perspectives on Academic Careers—Facilitators and Challenges. LGBT Health, 2(4), 346–356. https://doi.org/10.1089/lgbt.2015.0024

7e: Conroy, M. L., Wilkins, K. M., van Dyck, L. I., & Yarns, B. C. (2022). *Geriatric psychiatry across the spectrum: Medical student, resident, and fellow education*. **Psychiatric Clinics of North America, 45**(3), 381–395. https://doi.org/10.1016/j.psc.2022.07.008

8e: Vaughn, R. L., Morris Smith, L., Bernstein, C. A., Hansen, H., Ofori-Atta, A., & Ohene, S. (2016). Expanding the Pipeline: The New York University School of Medicine–University of Ghana School of Medicine and Dentistry Psychiatric Education Initiative. International Journal of Mental Health, 45(2), 154–159. https://doi.org/10.1080/00207411.2016.1167490

9e: Yager, J., Waitzkin, H., Parker, T., & Duran, B. (2007). Educating, Training, and Mentoring Minority Faculty and Other Trainees in Mental Health Services Research. Academic Psychiatry, 31(2), 146–151. https://doi.org/10.1176/appi.ap.31.2.146

Supplementary material 2 – Studied included in this review

N° Reference	Country	Research type	Population (n/%)	Assessment	Intervention (yes/no)	Results
[27]	UK (London)	Qualitative research	Nursing students (n=15)	Semi-structured interviews and Focus Groups	**Yes** (Well-being workshop using the "Five Ways to Wellbeing" model)	Six main themes emerged, including: "What we brought," "Conceptualization," "Adjustment," "Giving them the tools," "What we gained," and "Development." Students gained peer facilitation skills and improved their well-being.
[18]	USA	Mixed-methods	Medical students (n=225) (M=50.5%, F=47%, Other=2.5%)	Focus groups and Surveys	**Yes** (Peer Advocate support)	The Peer Advocate program was used by a quarter of students. Most reported perceived benefits. Key areas addressed: relationship difficulties, mental well-being, and academic stress, including support for experiences of discrimination.
[16]	Norway	Cross-sectional study, psychometric validation	458 nurses with clinical tutoring experience	MCI and CFA	**No**	Validated the Norwegian version of the Mentor Competence Instrument (MCI). Results confirmed good construct validity and excellent internal consistency, making it a solid tool for evaluating nurse mentors.
[29]	USA	Mixed-methods	1st and 2nd year medical students	Pre/post course evaluation questionnaires and Focus groups	**Yes** (Senior Mentor Program pilot)	Improved attitudes toward aging and elderly care, plus increased geriatric assessment knowledge. Participants felt more enthusiastic and less afraid of interacting with the elderly.
[30]	USA	Mixed-methods	Nursing students	Video-recorded simulations with senior students and questionnaires	**Yes** (Simulations with junior mentees and senior peer mentor feedback)	Positive evaluations. Emerging themes: importance of watching recordings, value of repeated practice, deepening communication, and moving from intuition to goal-setting
[28]	UK	Mixed-methods evaluative study	Nursing students	Virtual tutorial sessions, Focus groups, interviews, and evaluation questionnaires	**Yes** (VIPS)	Reported positive results from engagement in a multi-institutional project. Highlighted the importance of a clear project vision for innovation and using technology for critical online discussions.
[31]	Spain	Mixed-methods descriptive evaluative study	Medical students (n=479)	Online forms, review meetings, academic performance analysis, and individual interviews	**Yes** (PSE-M)	Over 80% adherence. 40% identified with significant support needs; over a third utilized interventions for academic or emotional difficulties. Exceeded expectations for personalized support.
[32]	Serbia	Observational study with questionnaires	Medical students (n=858)	Questionnaire	No	67% of students reported continuous exposure to stressful situations. High stress factors included exam pressure, financial difficulties, family problems, and illness of a loved one.
.[33]	USA	Retrospective study	Medical students (n=39,316)	Statistical analysis of psychiatry/pediatrics match rates (Klingenstein Foundation schools vs. control)	**Yes** (Klingenstein Foundation medical mentorship programs)	Mentorship schools had a significantly higher match rate in psychiatry (6.1% vs 4.8%). Conversely, these schools had a slightly lower match rate in pediatrics.
[34]	Ireland	Qualitative	Psychiatric nursing students (n=6)	Semi-structured interviews	**Yes**	Positive experience for managing anxiety and integration. However, one student reported a negative experience due to prolonged mentor absence.
[35]	Australia	Retrospective observational	Medical residents (n=59)	DASS21, ProQOL, AUDIT	**No**	About half exceeded thresholds for depression/stress. Barriers to help: lack of time, privacy fears, embarrassment, and career impact.

[36]	China	Cross-sectional	Medical student (n=325)	PHQ9, GAD7, AI-student version	**No**	High symptoms of depression/anxiety. Mentoring quality mediated the relationship between research self-efficacy and emotional symptoms.
[37]	Australia	Qualitative	Registered nurse preceptors (n=13)	Focus groups	**Yes** (ICLM placement model)	Model offers flexibility but complicates mentoring; preceptors found it harder to evaluate students and build deep relationships.
[38]	Sweden	Qualitative	Preceptors (n=17); Nursing students (n=11)	Focus groups and qualitative analysis	**No**	Peer learning in psychiatry is interactive; mutual support and solid relationships foster safety, independence, and knowledge.
[57]	USA	Mixed-method pilot	Nursing students (n=23)	Error Choice Test, AQ-9, ANOVA	**Yes** (Direct contact mentorship)	Significant reduction in stigmatization. The model is effective for reducing stigma toward mental illness, though the AQ9 scale showed no change.
[39]	Spain	Qualitative	Nursing students and academic tutors	Focus Groups and thematic analysis	**Yes** (Personal tutoring)	Tutoring is seen as a bridge between theory and practice and is fundamental for professional and personal development.
[40]	UK	Qualitative	Healthcare students (n=14)	Semi-structured interviews and thematic analysis	**No**	Value found in PATs for academic guidance and motivation. Issues: inconsistency in relationship quality and lack of availability.
[41]	Germany	Cross-sectional survey	Medical students (n=543)	PH-4, teaching evaluation items, online survey	**No**	EMentoring participants showed better anxiety/depression levels than non-participants. Digital teaching was generally well-managed.
[42].	Quantitative	Medical students (n=162); M=115, F=47	Semi-structured questionnaire	Semi-structured questionnaire	**Yes** (Mentoring program)	Positive perceptions of both faculty and peer mentors. Barriers: scheduling difficulties, lack of time, and motivation.
[14]	USA	Descriptive mixed-method	Medical students (n=27)	Structured survey and qualitative analysis	**No**	60% inclined toward academic careers. Barriers: poor knowledge of academic paths and stereotypes about the elderly.
.[43]	Iran	Descriptive evaluative	Junior students (n=371); Senior students (n=10)	Online mentoring and questionnaire	**Yes** (Virtual peer mentoring pilot)	Social platform helped students adapt to emergency conditions. Seniors felt professional benefits from teaching.

[44]	UK	Qualitative	Nursing students (n=50); Mentors (n=7)	Clinical evaluation, focus groups, thematic analysis	**Yes** (PACS model)	Supervisors felt valued. Students initially uncertain but eventually recognized the value of patient-centered supervision.
[45]	Canada	Qualitative	Nursing students (n=7); Prof. Nurses (n=7)	Interviews and 3-month follow-up	**Yes** (4 follow-up sessions)	.<br>Increased professional self-awareness. Recreational activities improved dialogue and the sense of belonging.
[46]	Spain	Mixed-method	Nursing students (n=163)	10-point Likert questionnaire and Focus groups	**No**	Mentors rated highly (8.43/10). Learning-by-doing was most effective. Mental health and emergency placements received the highest scores.
[47]	USA	Descriptive mixed-method	Nursing students (n=213)	Electronic survey and thematic analysis	**No**	55.2% received peer mentorship. Benefits: connection, research skills. Barriers: time, lack of institutional support, and poor matching.
[48]	UK	Descriptive	Nursing students (n=1425)	Closed-response questionnaire, thematic analysis, interviews	**No**	Students often perceived unfair treatment. A layered mentorship model was designed to improve clinical support.
[49]	Japan	Qualitative	Faculty (n=9)	Semi-structured interviews and qualitative analysis	**No**	Mentoring is complex and vital. Need for more training, structured resources, and guidelines for qualitative research PhD paths.
[50]	UK	Qualitative	Nursing students (n=34)	Questionnaire and open-response analysis	**No**	Suggests strengthening mentor support and including specific mental health skills and multidisciplinary competencies.
[51]	USA	Cross-sectional mixed-method	Medical students (n=201)	Online questionnaire and quantitative analysis	**No**	Pandemic hindered ENT training; digital solutions (online mentorship) partially compensated for clinical losses.
[52]	Brazil	Descriptive observational	Medical students in difficulty (n=33)	Online tutor questionnaire and 2-year follow-up	**Yes** (Structured intervention)	72% overcame difficulties and continued their studies. Tutors provided socioeconomic and emotional support. Institutional recognition is needed.
[53]	UK	Qualitative	Mentors (n=29)	Semi-structured Focus Groups	**No**	Placement planning depends heavily on human resources; decision-making is poorly structured. Needs stronger university-service collaboration.

[54]	USA	Qualitative/Evaluative	LTC nursing students	Interactive training and post-program evaluation	**Yes** (ECLEPs program)	Facilitated collaboration between LTC nurses and faculty; improved student experience and integration into facilities.
[55]	UK	Qualitative	University faculty (n=3)	Semi-structured interviews	**No**	Faculty familiar with support procedures but face barriers like bureaucracy and ambiguous roles in coordinating adjustments.
[56]	USA	Mixed-method	252 LGBT professionals and trainees	Questionnaire and focus groups	**No**	Absence of LGBT role models and fear of discrimination are barriers. LGBT mentors are key for identity, support, and career resilience.
[11]	USA	Descriptive	Pre-doctoral students and mentors	Qualitative evaluation	**Yes** (Structured program)	Formalized access for HBCU students to Ivy League resources. Promoted African American leadership in clinical research (HIV/infections).
[12]	Netherlands	Mixed-method	Final-year nursing students	Qualitative analysis and participatory reflection	**Yes** (Students as co-researchers)	Involvement as co-researchers fostered deep learning, improved relational skills, and social awareness.
[58]	USA	Quantitative	Medical students (n=30)	Likert scale surveys	**Yes** (START-MH program, 10 weeks)	Increased enthusiasm and interest in research careers. Students evaluated the intensive geriatric mentoring positively.
[59]	Brazil	Comparative (Exp vs Control)	2nd-year medical students (n=95)	WHOQOLBREF, DASS21, AMS	**Yes** (Longitudinal mentorship)	No statistically significant effects on quality of life, mental health, or motivation compared to control.
[60]	USA	Descriptive	Med students (n=900); Mentors (n=600)	Qualitative and quantitative evaluation	**Yes** (Student-mentor matching)	Virtual support was crucial for students from low-resource institutions during periods of isolation and uncertainty.
[61]	USA & Canada	Mixed-method	93 Medical Universities	Semi-structured questionnaire; qualitative/quantitative analysis	**No**	80% have procedures for unprofessional behavior. Weaknesses: lack of faculty training, non-uniform policies, and lack of efficacy evaluation.
[62]	Finland	Qualitative	Nursing students (n=23)	Data collection through observation	**Yes** (Group supervision)	Group supervision was a positive alternative to 1-on-1. The staff nurse mentor role was crucial for understanding professional concepts.
[63]	Brazil	Cross-sectional	Med students (n=953); M=20%, F=78%, NB=1%	24-question survey	**No**	High episodes of gender discrimination, especially for women and non-binary students. Very few had access to a mentor.

[64]	USA	Descriptive	Medical students	No evaluation listed	**Yes** (Fireside Chats)	"Fireside Chats" offered an informal space for emotional support and sharing thoughts during the pandemic.
[65]	Canada	Qualitative	Medical residents (n=11)	Semi-structured interviews	**Yes** (Cascading mentorship)	Improved CanMEDS competencies (communication, leadership). Residents found the mentor role rewarding.
[66]	UK	Qualitative	Nursing students (n=7); Faculty (n=3)	Semi-structured interviews	**No**	Identified three themes: defining modifications, support methods, and professional tasks. Process is complex and influenced by many factors.
[67]	UK	Qualitative	6 tutor-student pairs	Audio recordings of 12 meetings and DNA analysis	**Yes** (Personal tutor meetings)	Tutors helped students overcome "perfectionism" and focus on self-care. Relationship enriched when tutors shared personal experiences.
[68]	Canada	Qualitative	Nursing students (n=11)	Semi-structured interviews	**Yes** (Peer mentorship program)	Helpful behaviors identified at academic, social, professional, and mental levels. Significant personal growth noted.
[69]	France	Qualitative	Medical students	Semi-structured interviews (IPA)	**No**	Intense formative experience; changed perception of mental health. Highlighted need for careful tutoring given student vulnerability.
[70]	UK	Descriptive	No numbers reported	Description and reflection on practice	**Yes** (Peer mentoring intervention)	Provides a practical view of peer mentoring in nursing, focusing on reflexivity and professional development in real contexts.
[71]	Canada	Qualitative	Nursing students (n=8); Mentors (n=3)	Reflective collaborative writing	**Yes** (Mentorship at QHR conference)	Created an authentic learning community. Boosted confidence, sense of belonging, and recognition as emerging researchers.
[72]	UK	Quantitative	Medical students (n=53); F=56.3%, M=43.7%	Pre/post online questionnaires, Likert scale, t-test	**Yes** (Peer-led mixed-approach sessions)	Significantly increased perceived confidence, preparation, and clinical performance. High satisfaction with remote format.
[73]	USA	Descriptive	Recruited students (n=26); 85% F, 69% AA, 23% H/L	Surveys, focus groups, structured interviews	**Yes** (HIV Vaccine Research & Mentorship)	Strengthened academic skills and motivation. Access to role models impacted professional identity and career ambitions.
[74]	USA	Qualitative	Medical students (n=13)	Semi-structured interviews and qualitative analysis	**Yes** (National child psychiatry mentoring)	Increased awareness and interest in the field. Improved clarity of career goals and sense of belonging.

[75]	Australia	Qualitative	Medical students (n=37)	Focus groups and thematic analysis [268]	**No**	Recognized stigma in the profession. Requested practical, humanizing teaching through role models and reflective spaces.
[77]	USA	Descriptive	Nursing students (n=90)	Post-survey and quantitative analysis	**Yes** (Peer mentoring program)	Promoted connection and support. Mentees felt more motivated; mentors developed leadership and communication skills.
[78]	Canada	Descriptive	Medical students (n=60)	Pre/post questionnaire	**Yes** (Mentorship program)	Beneficial for personal well-being and professional development. Improved career satisfaction and specialty definition.
[10]	USA	Qualitative	Nursing students & mentors (n=70)	Qualitative focus groups	**Yes** (Direct mentorship program)	Strong perceptions of stigma by health workers. Anti-stigma messages and mentor selection are key structural elements for impact.
[79]	India	Observational	1st-year med students (n=150); Mentors (n=12)	Semi-structured questionnaire	**Yes** (Virtual mentoring program)	Counteracted loneliness and facilitated connection. Highlighted need for better technological tools.
[80]	UK	Qualitative	Nursing students (n=15)	Focus groups and thematic analysis	**Yes** (C-PAL coaching model)	Positive view of peer support for confidence. Critical factors: insufficient preparation, competition between students, and shift organization.
[81]	Finland	Cross-sectional	Nursing and midwifery students (n=2609)	Online CLES+T questionnaire	**No**	Assigned mentors, clear goals, and good theory-practice links correlate with the most positive clinical environment perceptions.
[82]	Japan	Longitudinal	8,262,000 students (1989-2012)	Annual surveys	**No**	Suicide is the leading cause of death (40%+). Risk higher for males, medical students, and final-year students. Minority had mental health contact.
[83]	USA	Qualitative	Nursing students (n=42)	ATP-30 and AMI	Yes (Pre-clinical psychiatry course)	Significant improvement in attitudes toward psychiatry (ATP-30) but not toward mental illness (AMI).
**[84]**	USA	Longitudinal	Medical students (n=286); M=28%, F=47%	Annual questionnaires and logistic regression	**No**	Debt negatively influenced primary care choice. Family background and finances were key determinants for specialty choice.
[85]	UK	Qualitative	Newly graduated nursing students (n=12)	Semi-structured interviews	**No**	Transition is difficult; low confidence in complex clinical situations. Mentoring quality is essential for professional entry.

[86]	UK	Qualitative	Students (n=12), Mentors (n=5), Faculty (n=6)	Semi-structured interviews	**No**	Education fosters professional integrity and boundary recognition, helping students address barriers to reporting issues.
[87]	Canada	Descriptive	Medical students (n=50)	Pre/post questionnaire and Likert scale	**Yes** (Cascading mentorship)	Increased confidence in advocacy and adolescent development knowledge. Enriched mentoring for LGBTQ+ youth.
[88]	Taiwan	Retrospective cohort	Medical students (n=65)	Knowledge and OSCE clinical competence	**No**	High technical knowledge linked to high clinical competence but also high burnout. Non-technical attributes associated with lower burnout.
[89]	USA	Qualitative	Medical students (n=3)	Qualitative observation	**Yes** (8-week summer program)	Strengthened research curiosity and confidence. Offered a concrete view of academic psychiatry opportunities.
[90]	USA	Mixed-method	Nursing students (n=12); Mentors (n=9)	Post-intervention Likert questionnaire	**Yes** (Virtual peer mentoring)	Increased support, confidence, and adaptation for mentees. Mentors improved communication and leadership. Desired hybrid format.
[91]	UK	Mixed-method	Nursing and midwifery students (n=215)	Questionnaires and EHR access	**No**	Only half had placement EHR access. Barriers: lack of specific training, mentor unfamiliarity with systems, and unclear procedures.
[9]	Canada	Descriptive	Medical students (n=21); M=8, F=13	SurveyMonkey questionnaire	**Yes** ("Doc to Do" peer mentoring) [364]	In-person contact improved perceived efficacy over email/messaging. Critical issues: lack of structure and regular contact.
[92]	Hungary	Qualitative	Medical students (n=17); M=7, F=10	Semi-structured interviews	**Yes** (Near-peer mentoring)	Mentors felt enriched personally and professionally. Improved communication/leadership. Challenges: time management and expectations.
[93]	France	Methodological	10 experts (internship tutors)	Delphi Method	**No**	Developed a 68-item internship register for psychiatric nursing. Adaptable for tracking competence via self and external evaluation.
[94]	Turkey	Qualitative	Nursing students (n=14); M=5, F=9	Individual interviews	**No**	International students reported language/culture barriers and isolation. Importance of academic/social support for integration.

Notes: (MCI) Mentor Competence Instrument; (CFA) Confirmatory Factor Analysis; (VIPS) **Virtual in Practice Support**; (PSE-M)Programa de Apoyo a los Estudiantes de Medicina; (OR); (PATs) Personal Academic Tutors; **Odds Ratio; (DASS-21)** *Depression Anxiety Stress Scales – 21 item version; (***ProQOL)** *Professional Quality of Life Scale; (***AUDIT)** *Alcohol Use Disorders Identification Test;* (PHQ-9) Patient Health Questionnaire-9; (GAD-7) Generalized Anxiety Disorder-7; **(**ICLM) **Integrated Clinical Learning Model;** (**AQ) Attribution Questionnaire;** (PH-4) *Patient Health Questionnaire-4;* (LTC) Clinical Education Liaisons; (ECLEPs)Enhanced

Clinical Learning Environments in Long-Term Care Partnership; (**START-MH)** Summer Training on Aging Research Topics – Mental Health; **(WHOQOL-BREF)** *World Health Organization Quality of Life – BREF version***; (AMS)** *Academic Motivation Scale;* **(DNA) dialogical narrative analysis; (IPA) Interpretative Phenomenological Analysis;** (C-PAL) Coaching and Peer-Assisted Learning; (CLES+T) Clinical Learning Environment, Supervision and Nurse Teacher; (**ATP-30***)(Attitudes Toward Psychiatry – 30 items*; (**AMI)** *Attitudes to Mental Illness questionnaire*;(OSCE) Objective Structured Clinical Examination;(EHR) **Electronic Health Record.**
